# On the Use of Quinoidine as a Substitute for Quinia in Miasmatic Fevers

**Published:** 1857-05

**Authors:** B. F. Graham

**Affiliations:** Wake Co., N. Carolina


ART. III.—*On the Use of Quinoidine as a Substitute for Quinia in Miasmatic Fevers.* By B. F. GRAHAM, M.D., Wake Co., N. C.

IN the first issue of the "North American Med. Chir. Review," the subject of the "Economical Use of Quinia in Malarious Fevers," by O. C. Gibbs, M.D., is presented to the consideration of the medical profession. Looking, as he justly remarks, at the high price of the article, the probable failure, at no distant day, of the cinchona tree, and also the increasing consumption of quinia in other than miasmatic diseases,—should not these considerations alone stimulate the profession to put forth some exertion in search of a proper substitute?

Happily a most excellent substitute, as experience enables me to testify, is found in the concomitant chemical product, "Chinoidine or Quinoidine," for which, although we are dependent upon the same source as for quinia, yet, by its general substitution, particularly in malarious fevers, the consumption of the latter would be materially economized. Since the publication of a report on the use of quinoidine, by J. Da Costa, M.D., in the Medical Examiner for May, 1855, I have used this remedy in the above fevers, with a success beyond my most sanguine expectations.

Finding many physicians, some of whom have not read that or any other report on its use, and others who have neglected to improve the valuable hints therein contained, I am induced, after fairly testing the utility of the remedy, to urge its claims, and if I shall succeed in prompting any of my professional brethren to direct their attention to its efficacy, my object will have been accomplished.

In promptness, I consider the quinoidine fully equal to quinia, and in permanency of cure, far superior. In proof of its efficacy, I will simply remark, that even in quotidian intermittents, I have often exhibited the medicine with the happy effect of cutting short the disease, without the recurrence of a single paroxysm; and in several instances, after quinia, in large quantities, had been administered without the desired result, I have, by the use of quinoidine alone, succeeded in effecting a speedy and permanent cure. Nor do I now recollect a single instance in which, after its exhibition, a relapse occurred, whereas the experience of almost every practitioner, will but too amply corroborate my own in regard to the common occurrence of relapses after the use of quinia.

Besides its comparative cheapness, there are other considerations that should give quinoidine a precedence over sulphate of quinia. It fails to produce those unpleasant and sometimes deleterious effects, such as headache, tinnitus aurium, dimness of vision, &c., which so frequently attend the administration of the sulphate. Nor has the exhibition of the purified article, as prepared by Messrs. Powers and Weightman, ever been attended, in my experience, by the slightest nausea; but the unpurified, which is of the color and consistence of tar, not only excites nausea, but also active emesis, and if given in large doses, frequently repeated, may induce a dangerous hypercatharsis.

Quinoidine is also a good tonic, and in connection with iodine and ipecacuhana, an excellent alterative. But it is as an antiperiodic in malarious fevers, that I would more especially insist upon its claims, while I am disposed to believe, that after fairly testing the remedy, few physicians would be willing to lay it aside for the resumption of quinia.

I give it in pills of two grains each, every hour till the disease is arrested, and afterwards continue it for a few days, in the same doses, much less frequently repeated; the average quantity required for each patient being from thirty to forty grains.

I have also successfully administered the liquor pot. arsenitis, potassii iodidum, sodii chloridum, chloroform, &c., but do not think that any one of them is likely soon to become a permanent substitute for sulphate of quinia.

